# Characterization and Functional Analysis of *trehalase* Related to Chitin Metabolism in *Glyphodes pyloalis* Walker (Lepidoptera: Pyralidae)

**DOI:** 10.3390/insects12040370

**Published:** 2021-04-20

**Authors:** Zuo-min Shao, Jian-hao Ding, De-lei Jiang, Zhi-xiang Liu, Yi-jiangcheng Li, Jiao Wang, Jun Wang, Sheng Sheng, Fu-an Wu

**Affiliations:** 1 Jiangsu Key Laboratory of Sericultural Biology and Biotechnology, School of Biotechnology, Jiangsu University of Science and Technology, Zhenjiang 212018, China; shaozuomin23@163.com (Z.-m.S.); lostone1@126.com (J.-h.D.); 189310002@stu.just.edu.cn (D.-l.J.); 18852864112@163.com (Z.-x.L.); 192310019@stu.just.edu.cn (Y.-j.L.); 189310018@stu.just.edu.cn (J.W.); wangjun@just.edu.cn (J.W.); parasitoids@163.com (S.S.); 2 Key Laboratory of Silkworm and Mulberry Genetic Improvement, Ministry of Agriculture and Rural Affairs, Sericultural Research Institute, Chinese Academy of Agricultural Science, Zhenjiang 212018, China

**Keywords:** *Glyphodes pyloalis* Walker, *trehalase*, chitin metabolism, Validamycin A, RNA interference, pest control

## Abstract

**Simple Summary:**

Sericulture has always been threatened by *Glyphodes pyloalis* Walker (*G. pyloalis*). Trehalase is an essential enzyme in chitin metabolism and energy supply. In this study, two trehalase genes in *G. pyloalis* (*GpTre1* and *GpTre2*) were identified and functionally analyzed. Knockdown of the two genes led to the significant downregulation of chitin metabolism pathway-related genes, the difficulty in molting of larvae, and the deformation of adult wings. Moreover, the trehalase inhibitor, Validamycin A, treatment increased *GpTre1* and *GpTre2* expression and affected the expressions of chitin metabolism pathway-related genes. The inhibitor also caused a significantly increased cumulative mortality of larvae. The results suggested that *GpTre1* and *GpTre2* played a vital role on *G. pyloalis* development, which could be useful for providing information for insect pest control in the future.

**Abstract:**

*Glyphodes pyloalis* Walker (*G. pyloalis*) is a serious pest on mulberry. Due to the increasing pesticide resistance, the development of new and effective environmental methods to control *G. pyloalis* is needed. Trehalase is an essential enzyme in trehalose hydrolysis and energy supply, and it has been considered a promising target for insect pest control. However, the specific function of *trehalase* in *G. pyloalis* has not been reported. In this study, two trehalase genes (*GpTre1* and *GpTre2*) were identified from our previous transcriptome database. The functions of the *trehalase* in chitin metabolism were studied by injecting larvae with dsRNAs and trehalase inhibitor, Validamycin A. The open reading frames (ORFs) of GpTre1 and GpTre2 were 1,704 bp and 1,869 bp, which encoded 567 and 622 amino acid residues, respectively. Both of *GpTre1* and *GpTre2* were mainly expressed in the head and midgut. The highest expression levels of them were in 5th instar during different development stages. Moreover, knockdown both of *GpTre1* and *GpTre2* by the dsRNAs led to significantly decreased expression of chitin metabolism pathway-related genes, including *GpCHSA*, *GpCDA1*, *GpCDA2*, *GpCHT3a*, *GpCHT7*, *GpCHSB*, *GpCHT-h*, *GpCHT3b*, *GpPAGM,* and *GpUAP*, and abnormal phenotypes. Furthermore, the trehalase inhibitor, Validamycin A, treatment increased the expressions of *GpTre1* and *GpTre2*, increased content of trehalose, and decreased the levels of glycogen and glucose. Additionally, the inhibitor caused a significantly increased cumulative mortality of *G. pyloalis* larvae on the 2nd (16%) to 6th (41.3%) day, and decreased the rate of cumulative pupation (72.3%) compared with the control group (95.6%). After the activities of trehalase were suppressed, the expressions of 6 integument chitin metabolism-related genes decreased significantly at 24 h and increased at 48 h. The expressions of *GpCHSB* and *GpCHT-h*, involved in chitin metabolism pathway of peritrophic membrane in the midgut, increased at 24 h and 48 h, and there were no changes to *GpCHT3b* and *GpPAGM*. These results reveal that *GpTre1* and *GpTre2* play an essential role in the growth of *G. pyloalis* by affecting chitin metabolism, and this provides useful information for insect pest control in the future.

## 1. Introduction

The oligophagous silkworm is an economically important insect that eats mulberry leaves. The sericultural production plays a vital role in the economic income of many developing countries, such as China and India. Sericulture has always been threatened by insect pests, and these pests cause considerable financial loss every year [1,2]. *Glyphodes pyloalis* Walker (*G. pyloalis*) is one of the most serious insect pests on mulberry. *G. pyloalis* not only competes with silkworm for food but also spreads pathogens to silkworm larvae by contaminating mulberry leaves with the excrement [3]. In recent years, the quality and quantity of mulberry have been seriously threatened by *G. pyloalis* [4], and the most widely used control method is the use of chemical insecticides. However, it is more and more difficult to control *G. pyloalis* for its increasing resistance level to chemical insecticides [5]. Moreover, chemical insecticides also threaten silkworm larvae and cause economic loss [6]. Therefore, finding safer and more effective strategies to control *G. pyloalis* is urgent. As an environmentally friendly and effective method, biocontrol is being studied by many scientists [7], and study of the role of *trehalase* will provide a foundation for developing an effective method to control *G. pyloalis*.

Trehalose is a non-reducing disaccharide composed of two glucose molecules, and is widely found in the biological world. As the major “blood sugar” in insects, trehalose plays a crucial role in all developmental stages and physiological processes by regulating energy metabolism [8], such as energy storage, resistance to abiotic stress, supporting energy during flight activities, etc [9]. Trehalase (EC 3.2.1.28) is an important enzyme that catalyzes the hydrolysis of one trehalose molecule into two glucose molecules. There are two forms of trehalase, soluble trehalase (Tre1) and membrane-bound trehalase (Tre2), which have been identified and cloned in many insect species, including *Tribolium castaneum* (Coleoptera: Tenebrionidae) [10], *Bombyx mori* (Lepidoptera: Bombycidae) [11], *Aphis glycines* (Homoptera: Aphididae) [12], *Spodoptera exigua* (Lepidoptera: Noctuidae) [13], and *Helicoverpa armigera* (Lepidoptera: Noctuidae) [14]. Moreover, the two trehalase genes show obvious tissue specificity in insects. *Tre1* is mainly expressed in the integument and malpighian tubules, while *Tre2* is more highly expressed in the fat body and tracheae [15]. Therefore, they may have essentially differential functions in different organs. Thus, study of functions of *trehalase* can serve as an effective target for the development of new agents against insect pests [16].

It has been reported that trehalase not only supplies energy, but also regulates the expressions of chitin metabolism-related genes in insects, and the suppression or knockdown of *trehalase* expression could inhibit chitin biosynthesis [10,17]. *Spodoptera exigua* showed significantly higher mortality rates after knockdown of *SeTre-1* and *SeTre-2*, and the expressions of chitin synthase gene A (*SeCHSA*) and chitin synthase gene B (*SeCHSB*) were inhibited after knockdown of *SeTre-1* and *SeTre-2*, respectively [15]. The wing development of *Nilaparvata lugens* (Homoptera: Delphacidae) was affected by *trehalase*, which could regulate the expressions of chitin metabolism and the wing development-related genes of wing bud tissue [18]. The development defects in *Harmonia axyridis* (Coleoptera: Coccinellidae) occurred after knockdown of *TRE2-like* and *TRE2*, especially in the ecdysis stage, and the expressions of *HaCHSA* and *HaCHSB* also significantly decreased [19]. As the first substrate in the chitin metabolism pathway in insects, trehalase can be used as an effective target for pest control [20].

Validamycin A has been considered a promising competitive trehalase inhibitor and useful tool for studying the function of trehalose metabolism in insects [16,21]. It has been widely used to control rice sheath blight in rice-producing areas for its inhibition of trehalase activity [22], and this inhibition could affect feeding, growth, development, and metabolism in insects [23,24,25]. After treatment with Validamycin A, there was an impediment in the growth of *Helicoverpa armigera*, developmental defects, and even a decrease in fecundity [16]. Validamycin A harms the development of *Aedes aegypti* (Diptera: Culicidae) larva, pupa, and the flight of adult [26].

Although the function of the *trehalase* has been studied in many other species, but the function of *trehalase* in *G. pyloalis* chitin metabolism has not yet been reported. Studying the *trehalase* will benefit the control of *G. pyloalis*. In this study, *GpTre1* and *GpTre2* were identified from the *G. pyloalis* transcriptome database (SRR13051671, SRR13051668, and SRR13051665) [27]. The functions of *GpTre1* and *GpTre2* were studied using RNAi and trehalase inhibitor.

## 2. Materials and Methods

### 2.1. G. pyloalis Raring and Samples Preparation

*G. pyloalis* were maintained since 2014 in the Key Laboratory of Silkworm and Mulberry Genetic Improvement, Ministry of Agriculture, Sericultural Research Institute, Chinese Academy of Agricultural Science. The larvae were reared on fresh mulberry leaves in an insect-rearing room with the conditions of 25 ± 1 °C, 60–80% relative humidity, and a 14 h light and 10 h dark photoperiod.

The phases of *G. pyloalis* larvae have been classified into five instars based on the morphological features of the insect and the number of molting times. The individual genetic differences were minimized by mixing 30 samples for detecting tissue expression pattern. The head, integument, midgut, fat body, and hemolymph from the second day of fifth instar larvae were dissected, cleaned with DEPC water (RNAase-free), and stored at −80 °C for further use. Three replicates of each experiment were performed.

### 2.2. Bioinformatics Analysis of GpTre1 and GpTre2

The ORFs of putative *GpTre1* and *GpTre2* genes were predicted by ORF finder (https://www.ncbi.nlm.nih.gov/orfnder/ (accessed on 21 February 2020). The theoretical isoelectric point (*p*I) and molecular weight (MW) of each enzyme was predicted by ExPASy (https://web.expasy.org/compute_pi/) (accessed on 21 February 2020). Multiple alignments of various protein sequences were performed by DNAMAN 8.0 software (Lynnon Corporation, Quebec City, QC, Canada). Conserved motifs were predicted by online SMART software (http://smart.embl-heidelberg.de/) (accessed on 21 February 2020). The transmembrane helices were analyzed using TMHMM v.2.0 (http://www.cbs.dtu.dk/services/TMHMM-2.0/) (accessed on 21 February 2020). The signal peptide was predicated by SignalP-5.0 (http://www.cbs.dtu.dk/services/SignalP/) (accessed on 21 February 2020), and the N-glycosylation sites were analyzed by NetNGlyc 1.0 Server (http://www.cbs.dtu.dk/services/NetNGlyc/) (accessed on 21 February 2020). Phylogenetic analysis was conducted by MEGA-X software using the neighbor-joining method with 1,000 bootstrap replications. The protein sequences of GpTre homologs in 17 other species were downloaded from GenBank (http://www.ncbi.nlm.nih.gov/) (accessed on 11 March 2020). GenBank IDs of each protein are listed in Table S1.

### 2.3. RNA Isolation and cDNA Synthesis

Total RNA was isolated using TRIzol reagent (Invitrogen, New York, NY, USA), following the manufacturer’s instructions. RNA integrity was analyzed by electrophoresis with ethidium bromide staining, using 1% agarose gels. A Nanodrop 2000 spectrophotometer (Thermo Fisher Scientific, New York, NY, USA) was used to detect the RNA concentration and purity at absorbance ratios of A_260/230_ and A_260/280_. The PrimeScript^TM^ RT Reagent Kit with a gDNA Eraser (TaKaRa Biotechnology Co. Ltd., Dalian, China) was used to synthesize the first strand of cDNA according to the manufacturer’s instructions. In brief, reaction was incubated at 37 °C for 15 min and then 85 °C for 5 s in a 20 μL reaction system. The qualified cDNA was preserved at −20 °C for later use.

### 2.4. RT-qPCR Analysis of GpTre1 and GpTre2 Expression Levels

RT-qPCR was performed to analyze the expression patterns of *GpTre1* and *GpTre2*. The specific primers were designed by NCBI Primer-BLAST software (https://www.ncbi.nlm.nih.gov/tools/primer-blast/) (accessed on 26 February 2020) and are shown in Table S2. The LightCycler^®^96 PCR Detection System (Roche, Basel, Switzerland) was used to perform the reaction, which contained 7.5 μL of 2 × ChamQ SYBR qPCR Master Mix (Vazyme Biotech Co., Ltd., Nanjing, China), 0.6 μL of each gene-specific primer (0.4 μM), 4.8 μL of RNase free H_2_O, and 1.5 μL of cDNA. The reactions cycling profile were performed at 95 °C for 5 min, and 40 cycles at 95 °C for 20 s and 60 °C for 60 s. The relative expression level of each gene was calculated using the 2^−ΔΔCt^ method. *G. pyloali ribosomal protein L32* (*GpRpl32*) [28] as the reference gene was used for correction of the expression of *GpTre1* and *GpTre2*.

### 2.5. dsRNA Synthesis and Injection

Two specific dsRNAs targeting the functional domains of *GpTre1* and *GpTre2* (dsGpTre1 and dsGpTre2) were designed to ensure RNAi efficiency, respectively. Primers used to synthesize the dsRNA by Sangon Biotechnology (Shanghai, China) and are listed in Table S3. The dsRNA of green fluorescent protein (dsGFP) was used as a negative control. In vitro Transcription T7 Kit (for dsRNA synthesis; TaKaRa Biotechnology Co. Ltd., Dalian, China) was used to synthesize dsGpTre1, dsGpTre2, and dsGFP according to the manufacturer’s instructions. The integrity of the dsRNA was determined by 3% agarose gel electrophoresis. The qualified synthesized dsRNA was stored at −80 °C for later use.

RNAi was performed according to the previous method with some modifications [29]. In brief, dsGpTre1 and dsGpTre2 were dissolved in DEPC water to prepare a 2.0 μg/μL stock solution, respectively. Equal volumes of the two dsRNAs were mixed to obtain 1.0 μg/μL of the mixture, and 1.0 μL of the mixture was injected into each larva using the capillary needle with an inner diameter of 0.9–1.0 mm. One hundred twenty of the first day of 5th instar larvae were divided into four groups, each group contained thirty larvae, and treated with equal quantities of dsGFP, dsGpTre1, dsGpTre2, and a mixture of dsGpTre1 and dsGpTre2, respectively. Each group was replicated three times. Expression levels of *GpTre1* and *GpTre2* were analyzed using RT-qPCR at 24 h, 48 h, and 72 h after injection with dsRNA. The chitin metabolism-related genes involved in the integument and midgut peritrophic membrane were analyzed after RNAi of *GpTre1* and *GpTre2* at 24 h and 48 h, respectively.

The method used to investigate phenotypes and the mortality of *G. pyloalis* in different stages after RNAi were the same with the sample preparation as described above with some modifications. Ninety of the first day of 5th instar larvae were divided into three groups, and each group was treated with equal quantities of dsGFP, dsGpTre1, and dsGpTre1, respectively. The survival rate of larvae, pupation rate, and eclosion rate were counted, respectively. The changes of phenotype were also recorded every day. All treatments were performed for three biological replicates.

### 2.6. Analysis of the Effect of Validamycin A on GpTre1 and GpTre2 Expressions and Chitin Metabolism

To determine the effect of Validamycin A (Aladdin, Shanghai, China) on energy and chitin metabolism, the treatment was conducted on the first day of 4th instar larvae of *G. pyloalis* using the protocol previously described with some modifications [29]. In brief, 50 mg of farinose Validamycin A was dissolved in 10 mL of ddH_2_O to prepare a 5.0 μg/μL working solution. The ddH_2_O was used as a control. Sixty of the first day of 4th instar larvae were divided into two groups, and each group was injected with 1.0 μL of Validamycin A and ddH_2_O for each larva, respectively. Then, larvae were kept in the insect-rearing room, and the surviving larvae were collected 24 h, 48 h, and 72 h after injection for detection of the expression levels of *GpTre1* and *GpTre2.* Subsequently, the expressions of chitin metabolism-related genes involved in the integument and midgut pathways were analyzed at 24 h and 48 h, respectively, based on the expression patterns of *GpTre1* and *GpTre2*.

The method used to investigate phenotypes and the cumulative mortality of *G. pyloalis* in different stages after treatment with Validamycin A was the same as described above with some modifications. Briefly, fifty of the first day of 4th instar larvae were divided into two groups, and each group was treated with equal quantities Validamycin A and ddH_2_O for each larva, respectively. The cumulative mortality was counted from 1st–6th day, and the pupation rate was also recorded after treatment. The changes of phenotype were recorded every day. All treatments were performed for three biological replicates.

### 2.7. Determination of Trehalose, Glycogen, and Glucose Content

The trehalose, glycogen, and glucose content assay were conducted according to the method described by Leyva et al. [30], with some modifications. In brief, 5 surviving larvae collected 24 h and 48 h after treatment with Validamycin A was homogenized in phosphate buffer (pH 7.0), and the homogenate was centrifuged at 1000× *g* for 20 min at 4 °C. The supernatant was used for the measurement of trehalose, glucose, and glycogen. For trehalose, 10 mg of anthrone (Sinopharm, China) was dissolved in 10 mL of 98% sulfuric acid (Sinopharm, China) to prepare a working solution, and 30 μL of 1% sulfuric acid was mixed with an equal volume of sample at 90 °C for 10 min. 30 μL of 30% potassium hydroxide solution was added after the mixture was cooled on ice for 3 min, and then bathed at 90 °C for 10 min again. After cooling, 600 μL of anthrone working solution was added and bathed in 90 °C water for 10 min. Finally, the cooled sample was used for the detection of trehalose with an Infinite M200 Pro NanoQuant (TECAN, Männedorf, Switzerland) at 620 nm. The trehalose content was calculated based on the standard curve. For glucose and glycogen content measurements, the detection was performed by the Glucose Content Assay Kit and Glycogen Content Assay Kit (Sangon Biotech, Shanghai, China), according to the manufacturer’s instructions.

### 2.8. Statistical Analyses

The 2^−ΔΔCt^ method was adopted to calculate the relative expression level. A one-way analysis of variance (ANOVA), followed by Tukey’s post-hoc test, was used to analyze differences among three groups of data that met both normality and homogeneity of variance, while the Kruskal-Wallis test followed by Dunnett’s post-hoc test was used for data that did not meet normality. The *t*-test was used to compare two groups of data that satisfies normality and the Mann–Whitney test for data that do not meet normality. All data were analyzed using R version 4.0.0. A *p*-value < 0.05 was considered significant.

## 3. Results

### 3.1. Gene Identification and Sequence Analyses

**Figure 1:**
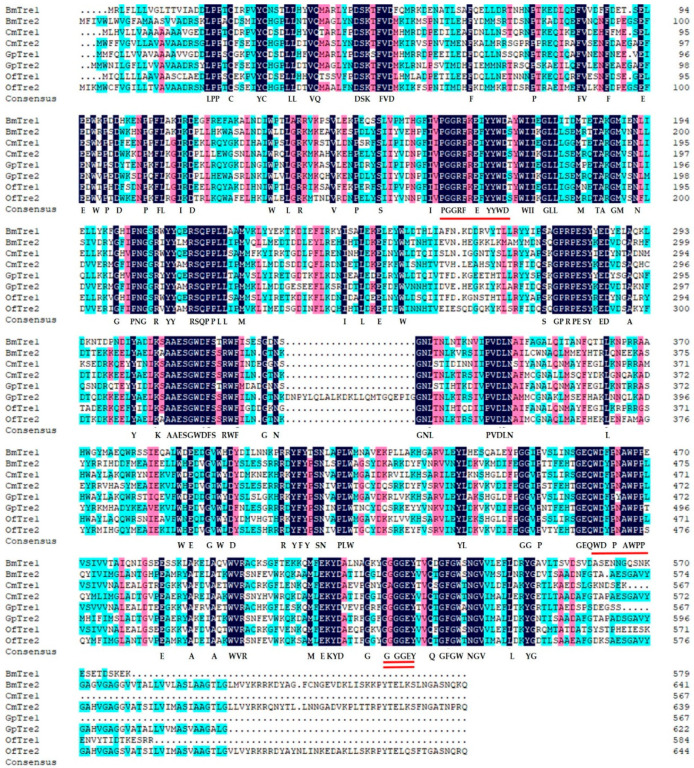
Amino acid sequences alignment of *G. pyloalis* trehalase with its homologues in other species using DNAMAN 8.0 software. Identical amino acids were highlighted in dark blue, and similar amino acids were highlighted in pink and aqua. Trehalase signature regions were indicated by the underline, and the glycine-rich region was indicated by the double underline. Bm, *Bombyx mori*; Cm, *Cnaphalocrocis medinalis*; Of, *Ostrinia furnacalis*. *BmTre1*, BAA13042.1; *BmTre2*, BAE45249.1; *CmTre1*, ALF03966.1; *CmTre2*, ANC68249.1; *OfTre1*, ANY30160.1; *OfTre2*, ANY30159.1.

*GpTre1* and *GpTre2* were identified from our previous transcriptome database of *G. pyloalis*, and they were uploaded to NCBI (accession numbers were MN915101 and MN915102, respectively) [27]. In this study, the cDNA sequence of GpTre1 contains an ORF of 1,704 bp encoding a protein of 567 amino acid residues with a predicted MW of 65 kDa and an isoelectric point (*p*I) of 5.01. The cDNA sequence of GpTre2 contains an ORF of 1,869 bp encoding a protein of 622 amino acid residues with a predicted MW of 71 kDa and a *p*I of 5.37. GpTre1 and GpTre2 have 4 potential N-glycosylation sites (amino acids 71, 76, 207, and 333) and 5 N-glycosylation sites (amino acids 46, 72, 258, 328, and 357), respectively (Figure S1). The predicted amino acid sequences of GpTre1 and GpTre2 contain two signature motifs, respectively (PGGRFREIYYWDTY, QWDFPYAWPP and PGGRFREFYYWDSY, QWDYPNAWPP) (Figure 1). Both have a glycine-rich region (GGGGEY) (Figure 1). Residues 1–17 and 1–16 are a signal peptide for GpTre1 and GpTre2, respectively (Figure S1). Additionally, GpTre2 contains a potential transmembrane domain (Figure S1), which is an important characteristic of Tre2.

**Figure 2:**
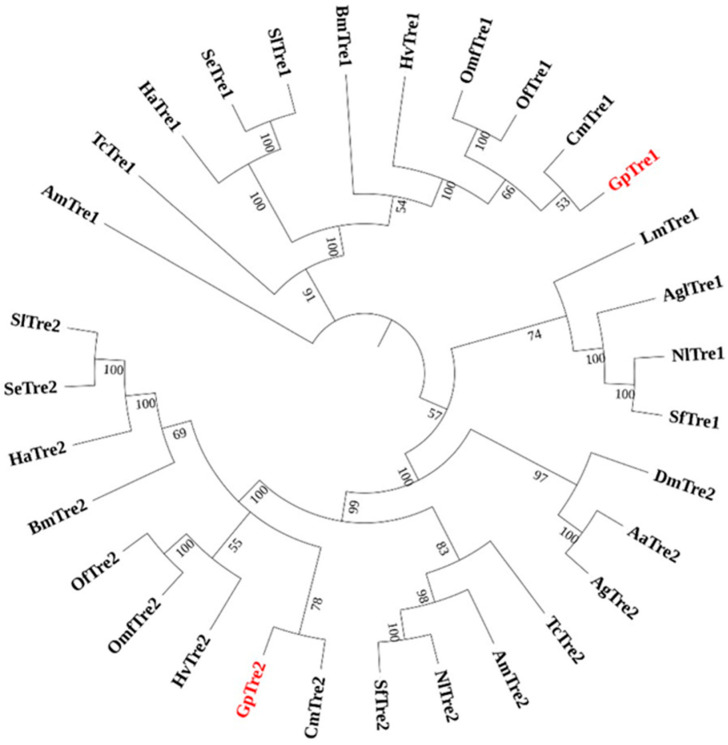
Phylogenetic analysis of trehalases in different species based on amino acid sequences. The evolutionary history was inferred using the neighbor-joining method with 1000 bootstraps, and next to the branches only percentages of replicate trees of above 50% are shown. *Aedes aegypti* (Aa), *Anopheles gambiae* (Ag), *Aphis glycines* (Agl), *Apis mellifera* (Am), *Drosophila melanogaster* (Dm), *Bombyx mori* (Bm), *Locusta migratoria manilensis* (Lm), *Nilaparvata lugens* (Nl), *Omphisa fuscidentalis* (Omf), *Spodoptera exigua* (Se), *Sogatella furcifera* (Sf), *Tribolium castaneum* (Tc), *Helicoverpa armigera* (Ha), *Heortia vitessoides* (Hv), *Spodoptera litura* (Sl), *Cnaphalocrocis medinalis* (Cm), *Ostrinia furnacalis* (Of).

BLASTP blast revealed that the GpTre1 protein sequence shared the highest identity with *Omphisa fuscidentalis* (Lepidoptera: Crambidae) (ABO20846.1, 76.16% identity), followed by *Cnaphalocrocis medinalis* (Lepidoptera: Pyralidae) (ALF03966.1, 75.74% identity). The GpTre2 protein sequence shared the highest identity with *Omphisa fuscidentalis* (ABO20845.1, 77.17% identity), followed by *Spodoptera exigua* (ABU95354.1, 72.67% identity). Multiple sequence alignments of trehalase proteins demonstrated a relative conservation of trehalase signature functions (Figure 1). Although the main catalytic domain and features of GpTre1 and GpTre2 were conserved with other trehalases, a sequence subtle change could lead to differential enzyme activities. Furthermore, the multiple sequence alignments and the sequence information indicated that GpTre1 is notably different from GpTre2 (Figure S1). The phylogenetic tree was constructed using MEGA-X software. A total of 31 trehalases from 18 species were grouped into 2 phylogenetic groups (Figure 2). Moreover, both GpTre1 and GpTre2 have a close evolutionary relationship with *Cnaphalocrocis medinalis*. The soluble trehalases were separately clustered from membrane-bound trehalases (Figure 2).

### 3.2. Spatiotemporal Expression Profiles Analysis of GpTre1 and GpTre2

**Figure 3:**
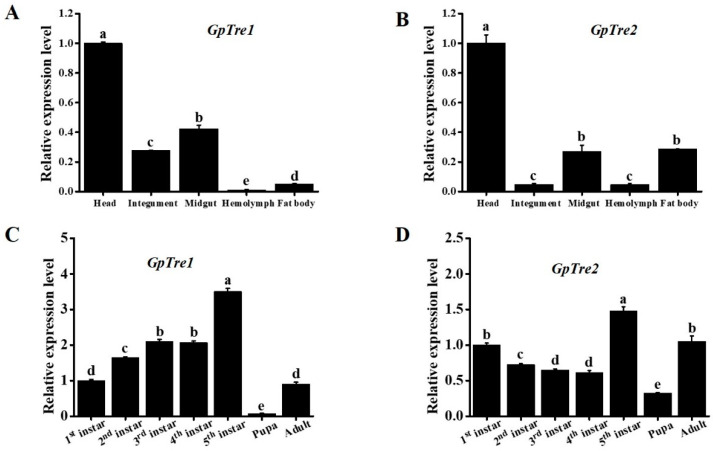
Analysis of the expression patterns of *GpTre1* and *GpTre2* in different development stages and tissues by RT-qPCR. (**A**,**B**) Different tissues from 5th instar larvae; (**C**,**D**) Different development stages samples were from the whole body. The *GpRpl32* gene was used as an internal reference gene. Data are shown as the mean ± SE of three biological replicates. The differences among different tissues were analyzed using non-parametric Kruskal–Wallis test followed by Dunnett’s post-hoc test. The differences among developmental stages were analyzed using one-way analysis of variance followed by Tukey’s post-hoc test. The different letters (a, b, c, d, and e) above the bars indicate significant differences (*p* < 0.05).

Expression patterns of *GpTre1* and *GpTre2* in different development stages of *G. pyloalis* were detected by RT-qPCR. The results showed that both *GpTre1* and *GpTre2* had significant differences in different development stages (df: 6; *F* values were 1593 and 282.9; *p* values were 2 × 10^16^ and 8.7 × 10^16^, respectively). The highest expression level of *GpTre1* and *GpTre2* was at 5th instar and the lowest at the pupa stage (Figure 3C,D). Moreover, *GpTre1* and *GpTre2* tissues expression patterns in the head, integument, midgut, fat body, and hemolymph were analyzed. The results revealed that *GpTre1* and *GpTre2* had significant differences in different tissues (df: 4; *p* values were 0.009 and 0.0152, respectively). The highest expression of *GpTre1* and *GpTre2* was in the head, followed by the midgut (Figure 3A,B). However, *GpTre1* had relatively high expression in the integument, while *GpTre2* was relatively high in the fat body.

### 3.3. Analysis of GpTre1 and GpTre2 Expressions and Phenotypes after RNAi

**Figure 4:**
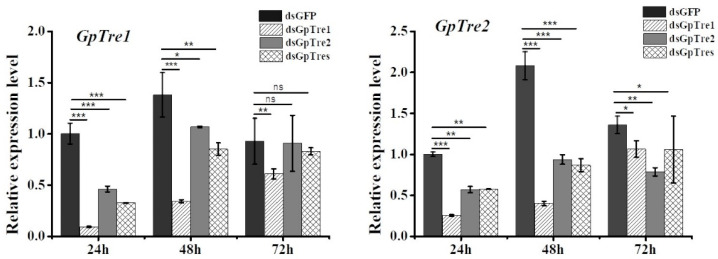
Analysis of the relative expression levels of *GpTre1* and *GpTre2* after injection with dsRNA. The first day of 5th instar larvae were selected for treatment. The *GpRpl32* gene was used as an internal reference gene. Data are shown as the mean ± SE of three biological replicates. Differences between each dsRNA and dsGFP group within each time point were compared using *t*-test. Asterisks above the bars indicate significant differences (* *p* < 0.05; ** *p* < 0.01; *** *p* < 0.001; ns, no significance difference).

To determine the role of *GpTre1* and *GpTre2* on *G. pyloali* development, RNAi was performed by injection of dsGpTre1 and dsGpTre2, respectively. The first day of 5th instar larvae were selected for treatment. The expression levels of *GpTre1* and *GpTre2* were detected 24 h, 48 h, and 72 h after injection with dsGpTre1, dsGpTre2, and a mixture of dsGpTre1 and dsGpTre2. dsGFP was used as the negative control. The results showed that three kinds of treatments injection with dsGpTre1, dsGpTre2, and the mixture of them all had a good effect on the expression of *GpTre1* and *GpTre2* at 24 h, 48 h, and 72 h, except for *GpTre1* at 72 h after injection with dsGpTre2 and the mixture (Figure 4). Among the three time points, relatively good RNAi effects were found at 24 h and 48 h, which were also selected for the next study (Figure 4).

**Figure 5:**
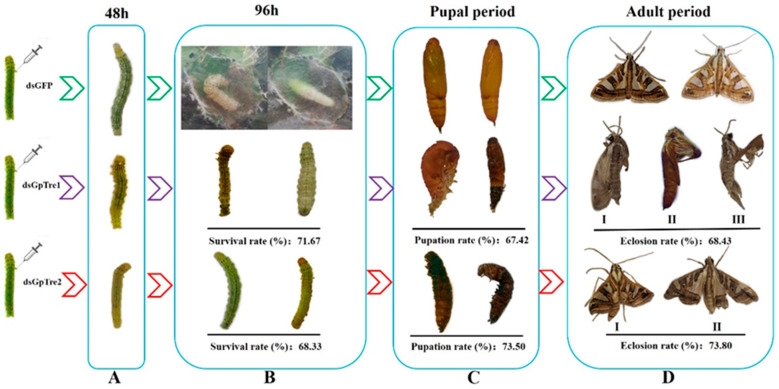
Representative phenotypes of *G. pyloalis* after injection with dsRNAs. (**A**) Larva, (**B**) prepupa, (**C**) pupa, and (**D**) adult.

According to the RNAi results of *GpTre1* and *GpTre2*, phenotypes among different development stages were investigated. Insects exhibited some abnormal phenotypes after knockdown of *GpTre1*, including slow growth, abnormal pupation, and serious deformities of the wings. Insects treated with dsGpTre2 exhibited similar phenotypes with the RNAi of *GpTre1*, except they were unable to fly even though they had relatively intact wings (Figure 5). These results showed that knockdown of *GpTre1* and *GpTre2* could be detrimental to *G. pyloali* development. The different phenotypes between the two treatments revealed that *GpTre1* and *GpTre2* have different functions in the development, especially in the adult stage (Figure 5).

### 3.4. Analysis of Chitin Metabolism-Related Key Genes after RNAi

**Figure 6:**
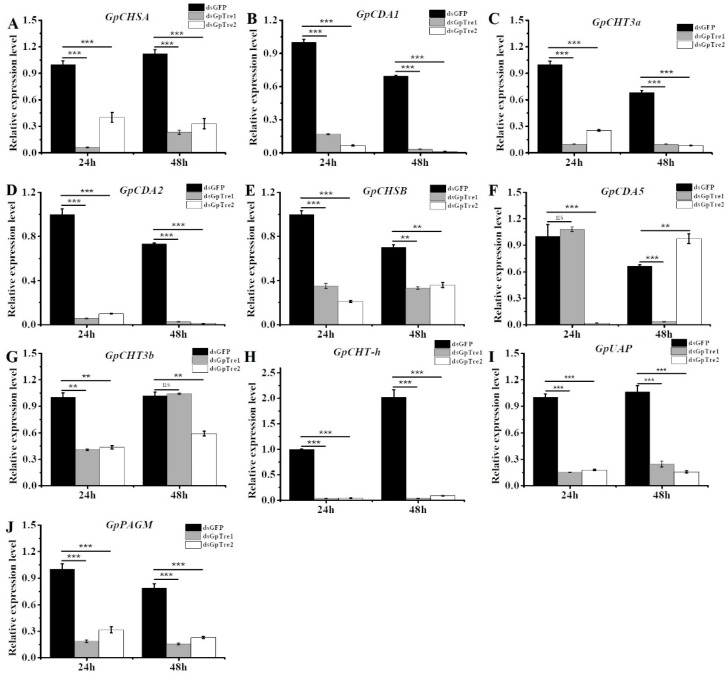
Analysis of the expression levels of key genes involved in the chitin metabolism pathway after knockdown of *GpTre1* and *GpTre2*. The expression levels were detected 24 h and 48 h after RNAi of *GpTre1* and *GpTre2* by RT-qPCR. (**A**–**D**) Expressions of 4 genes related to the integument pathway, (**E**–**H**) expressions of 4 genes related to the midgut pathway, (**I**,**J**) and the expressions of 2 key genes involved in chitin metabolism. dsGFP was used as the negative control. Data are showed as mean ± SE of three biological replicates. Differences between each dsRNA and dsGFP group within each time point was compared using non-parametric Kruskal–Wallis test followed by Dunnett’s post-hoc test. Asterisks above the bars indicated significant differences (** *p* < 0.01; *** *p* < 0.001; ns, no significance difference).

The expression levels of chitin metabolism pathway-related key genes belonging to the integument pathway and midgut pathway [27] were detected after knockdown of *GpTre1* and *GpTre2* by RT-qPCR. The results showed that the expressions of integument pathway-related genes, including *GpCHSA*, *GpCDA1*, *GpCDA2*, and *GpCHT3a*, were significantly downregulated 24 h and 48 h after knockdown of *GpTre1* and *GpTre2*, respectively (Figure 6A–D). Genes involved in the midgut pathway, including *GpCHSB*, *GpCHT3b*, and *GpCHT-h*, were also significantly downregulated 24 h and 48 h after injection with dsGpTre1 and dsGpTre2, respectively, while *GpCDA5* was downregulated after injection with dsGpTre2 at 24 h and after injection with dsGpTre1 at 48 h, and upregulated after injection with dsGpTre2 at 48 h (Figure 6E–H). Moreover, the expressions of two downstream key enzyme genes related to chitin metabolism, *GpUAP* and *GpPAGM*, were also detected. The results revealed that both *GpUAP* and *GpPAGM* decreased significantly 24 h and 48 h after injection with dsGpTre1 and dsGpTre2, respectively (Figure 6I,J).

### 3.5. Analysis of GpTre1 and GpTre2 Expressions after Validamycin A Treatment

**Figure 7:**
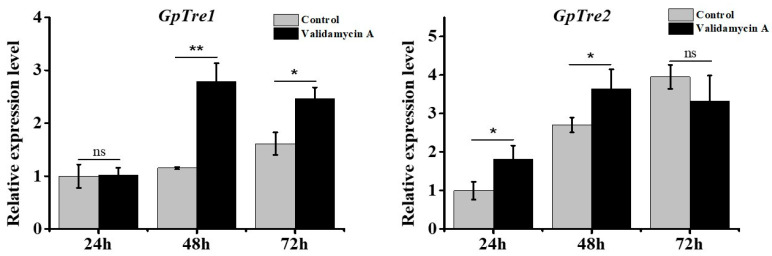
Changes in the expression levels of *GpTre1* and *GpTre2* at different times after injection with Validamycin A. The control group was treated with ddH_2_O. Data are shown as the mean ± SE of three biological replicates. Differences between Validamycin A and control group within each time point were compared using *t*-test. Asterisks above the bars indicate significant differences (* *p* < 0.05; ** *p* < 0.01; ns, no significance difference).

To further validate the roles of *GpTre1* and *GpTre2* in chitin metabolism, the expression levels of *GpTre1* and *GpTre2* were detected in 4th instar larvae after injection of Validamycin A at different times by RT-qPCR. The results indicated that the expression levels of *GpTre1* significantly increased 48 h and 72 h after treatment with Validamycin A. Moreover, *GpTre2* increased significantly 24 h and 48 h after treatment with Validamycin A (Figure 7).

### 3.6. Analysis of the Effect of Validamycin A on G. pyloalis Development

**Figure 8:**
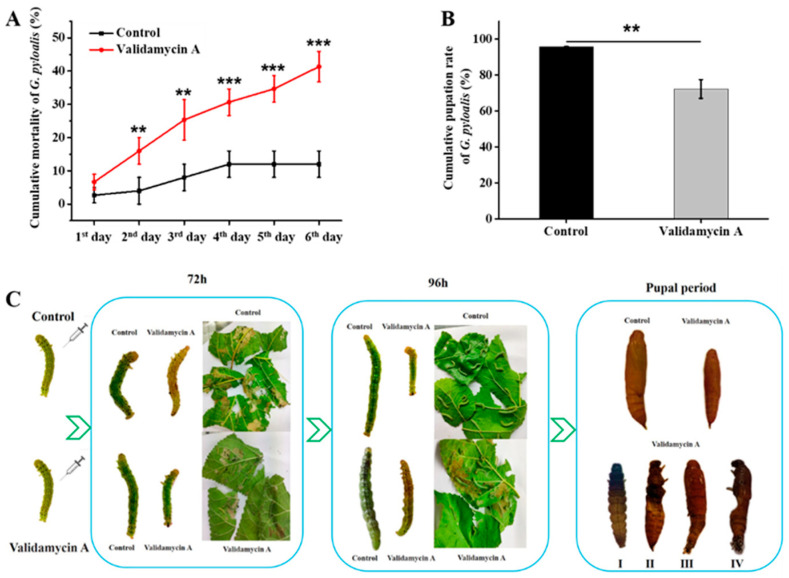
Analysis of the mortality and phenotypes after Validamycin A treatment. (**A**) Cumulative mortality, (**B**) cumulative pupation rate and (**C**) phenotypes of *G. pyloalis* after treatment with Validamycin A. The differences among three biological repeats were analyzed using R version 4.0.0 with one-way analysis of variance. All treatment group was compared with the control group at each time point. Asterisks above the bars indicated significant differences (** *p* < 0.01; *** *p* < 0.001).

The first day of 4th instar larvae were injected with 5.0 μg/μL of Validamycin A and used to investigate the cumulative mortality and phenotypes. The control group was injected with ddH_2_O. The results showed that the cumulative mortality of *G. pyloalis* larvae increased significantly on the 2nd (16%) to 6th (41.3%) day after injection with Validamycin A compared with the control (Figure 8A). Moreover, the cumulative pupation rate was 72.3%, which was significantly lower than the control group (95.6%) (Figure 8B). Furthermore, many larvae showed slow growth, abnormal molting, and pupation after Validamycin treatment (Figure 8C). These results revealed that *GpTre1* and *GpTre2* played an important role in energy and chitin metabolism.

### 3.7. Analysis of the Effect of Validamycin A on Carbohydrates

**Figure 9:**
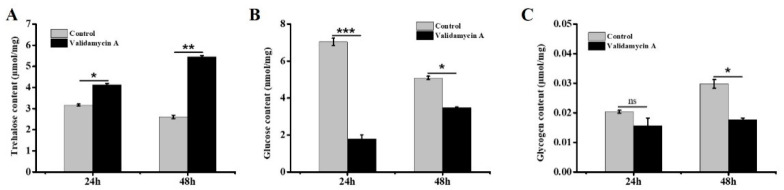
Detection of carbohydrate contents 24 h and 48 h after Validamycin A treatment. (**A**) Trehalose, (**B**) glucose, and (**C**) glycogen. Data are shown as the mean ± SE of three biological replicates. Differences between Validamycin A and control group within each time point were compared using non-parametric Mann–Whitney test. Asterisks above the bars indicated significant differences (* *p* < 0.05; ** *p* < 0.01; *** *p* < 0.001; ns, no significance difference).

According to the effects of Validamycin A on *GpTre1* and *GpTre2* expression as described above, the contents of trehalose, glycogen, and glucose were detected 24 h and 48 h after Validamycin A treatment. The trehalose content increased significantly 24 h and 48 h after treatment (Figure 9A), while the glycogen and glucose contents decreased significantly compared with the control (Figure 9B,C).

### 3.8. Validamycin A Treatment Significantly Affected the Expressions of Chitin Metabolism-Related Genes

**Figure 10:**
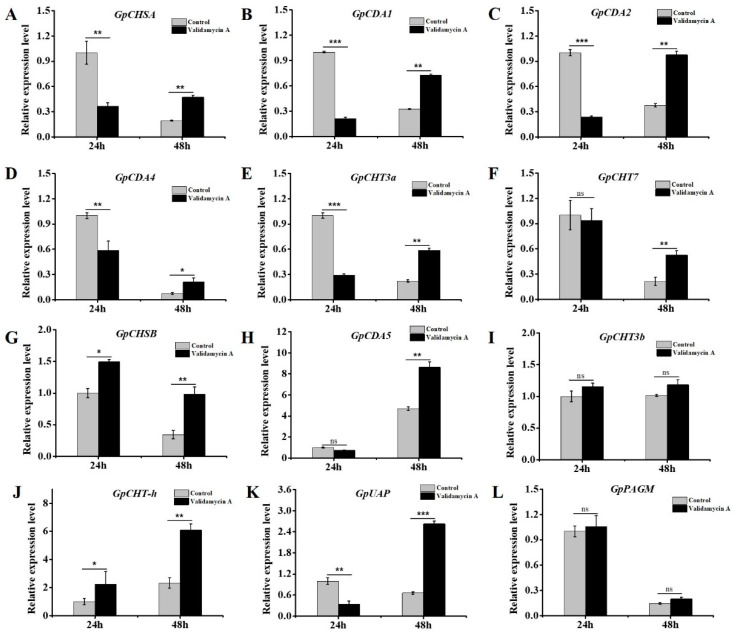
Analysis of the expressions of genes involved in the chitin metabolism pathway 24 h and 48 h after Validamycin A treatment by RT-qPCR. (**A**–**F**) Six genes involved in the integument pathway, (**G**–**J**) four genes involved in the midgut pathway, (**K**,**L**) and two key enzyme genes involved in chitin metabolism. The control group was injected with ddH_2_O. Data are shown as the mean ± SE of three biological replicates. Differences between Validamycin A and control group in (**B**,**C**) were compared using Mann–Whitney test, and others were compared using *t*-test. Asterisks above the bars indicated significant differences (* *p* < 0.05; ** *p* < 0.01; *** *p* < 0.001; ns, no significance difference).

To further validate the roles of *GpTre1* and *GpTre2* on chitin metabolism, the relative expression levels of chitin metabolism-related genes were detected by RT-qPCR. A total of 12 genes were selected and evaluated, including 6 genes (*GpCHSA*, *GpCDA1*, *GpCDA2*, *GpCDA4*, *GpCHT3a*, and *GpCHT7*) involved in the integument pathway, 4 genes (*GpCHSB*, *GpCDA5*, *GpCHT3b*, and *GpCHT-h*) involved in the midgut pathway and 2 key enzyme genes (*GpUAP* and *GpPAGM*) involved in chitin metabolism. The relative expression levels of *GpCHSA*, *GpCDA1*, *GpCDA2*, *GpCDA4*, *GpCHT3a*, and *GpCHT7* all decreased significantly at 24 h and increased significantly at 48 h after injection with Validamycin A (Figure 10A–F). *GpCHSB* and *GpCHT-h* increased 24 h and 48 h after injection (Figure 10G,J). *GpCDA5* increased 48 h after injection (Figure 10H), but *GpCHT3b* showed no obvious change after injection (Figure 10I). *GpUAP* showed downregulation at 24 h and upregulation at 48 h after treatment, but there was no significant change to *GpPAGM* (Figure 10K,L).

## 4. Discussion

Chitin is an essential component of cuticles and peritrophic membrane (PM) tissues, and it plays an important role in the regulation of insect growth and development. The processes of biosynthesis and degradation of chitin in insects are complex, and these dynamic processes are regulated by many kinds of enzymes. Many studies have revealed that the inhibition of these enzymes could be considered as a potential application for controlling insect pests. Trehalase is one of these, and it is highly valued for its important role in the chitin metabolism pathway of insects [31,32]. However, the function of *trehalase* in *G. pyloali* is still unclear. In this study, RNAi and Validamycin A were used to clarify *trehalase* function, which can lay a foundation for the control of *G. pyloali*.

Two *trehalase* genes, *GpTre1* and *GpTre2*, were identified from our previous transcriptome database. The two genes shared several common characteristics with little difference, including a signal peptide, two conserved signature motifs, and a highly conserved glycine-rich (GGGGEY) region (Figure 1). However, *GpTre2* has a transmembrane region, indicating these might be slightly functionally different, which could also be validated by the phylogenetic analysis (Figure 2) and the expression patterns in different tissues and development stages (Figure 3). This phenomenon could also be found in other species [33,34,35,36].

In this study, RNAi was used to study the function of the *trehalase* on the chitin metabolism of *G. pyloali*. The significant downregulation of *GpTre1* and *GpTre2* after treatment with dsGpTre1 and dsGpTre2, respectively, indicated a good interference effect of the dsRNA, and the two dsRNAs could be used for next study (Figure 4). After knockdown of *GpTre1* at 24 h and 48 h, four integument chitin metabolism pathway-related genes (*GpCHSA*, *GpCDA1*, *GpCDA2*, and *GpCHT3a*), four midgut pathway-related genes (*GpCHSB*, *GpCHT3b*, *GpCDA5*, and *GpCHT-h*), and two key enzymes (*GpUAP* and *GpPAGM*) were all basically downregulated (Figure 6), which could also be found in dsGpTre2 treatment group (Figure 6). These results suggested that *GpTre1* and *GpTre2* played a key role in chitin metabolism of *G. pyloali*, and these results were also consistent with previous reports in other species, such as *Nilaparvata lugens* [32,37,38]. To further validate the role of *GpTre1* and *GpTre2* in chitin metabolism, the trehalase inhibitor Validamycin A was used. The basically consistent expression patterns of 10 selected genes involved in the midgut and integument chitin metabolism pathways 24 h and 48 h after Validamycin A treatment (Figure 10) further validated the conclusion of RNAi that *GpTre1* and *GpTre2* were involved in the chitin metabolism. Moreover, the significant upregulation of selected genes 48 h after Validamycin A treatment (Figure 10) might be due to the involvement of other metabolic pathways by providing the substances needed by the chitin metabolic pathway. Furthermore, Validamycin A treatment led to the significantly increased content of trehalose (Figure 9A) and decreased glucose and glycogen content (Figure 9B,C) at 24 h and 48 h, and it may be that the glycogen was converted to glucose to compensate for the energy needed when trehalose could not serve as a source of energy.

Chitin is an essential component of integument and PM; the abnormality of chitin metabolism will obstruct the molting process, growth, and development of insects [16,18]. In this study, larvae showed slow growth, abnormal pupation, and defects to adult wings after knockdown of *GpTre1* and *GpTre2* (Figure 5), which was also found in *Laodelphax stritellus* (Homoptera: Delphacidae) [39], *Nilaparvata lugens* [32], *Tribolium castaneum* [10], and *migratory locust* [40]. The results revealed the essential roles of *GpTre1* and *GpTre2* in each stage of *G. pyloali* growth. Moreover, the serious wing deformities of adults caused by the effect of dsGpTre1 suggested that *GpTre1* might be a more suitable target for control pests than *GpTre2*. We speculated that the underlining mechanism of the phenotypes causing molting difficulty or failure to pupation was due to the inactivation of chitin biosynthesis or degradation-related genes. To further confirm this, *G. pyloalis* phenotypes in different development stages were analyzed after Validamycin A treatment (Figure 8). The significantly increased rate of cumulative mortality (Figure 8A) and decreased rate of pupation (Figure 8B), as well as the slow growth and abnormal molting and pupation (Figure 8C), further proved the effect of *GpTre1* and *GpTre2* on *G. pyloalis* growth by influencing the chitin metabolism pathway. This was also found in other species, such as *Drosophila* [41], *Leptinotarsa decemlineata* (Coleoptera: Chrysomelidae) [42], *Migratory Locust*, and *Chironomus riparius* (Diptera: Chironomidae) [43,44]. These results indicated that Validamycin A could inhibit the activities of two kinds of trehalases and lead to slow insect development and molting deformities by affecting chitin synthesis and degradation.

It is reasonable to conclude that *GpTre1* and *GpTre2* play an essential role in chitin synthesis and degradation processes by control of trehalose hydrolysis and energy supply. Moreover, the results in this study provide a foundation for the application of trehalase inhibitors or RNAi for pest control strategies.

## Supplementary Materials

The following are available online at https://www.mdpi.com/article/10.3390/insects12040370/s1, Table S1: Sequences and relevant information used for phylogenetic analysis of the trehalase genes, Table S2: Primers used to synthesize dsRNA, Table S3: Primers used in RT-qPCR, Figure S1: Characteristic analysis of GpTre1 and GpTre2 sequence. (A) and (B) are GpTre1 and GpTre2 nucleotide and deduced amino acid sequences, respectively. Underlines are potential N-glycosylation sites predicted by PROSCAN. The gray background is the amino acid sequence of the putative catalytic domain. The red letters are signature sequences that might be involved in catalytic function. Double underlines are signal peptides. The boxes are highly conserved glycine-rich regions. The blue letters are the putative transmembrane region predicted by TMHMM Server v. 2.0.
