# Supplementary material for: Characterization and Functional Analysis of trehalase Related to Chitin Metabolism in Glyphodes pyloalis Walker (Lepidoptera: Pyralidae)
Source: Insects. 2021 Apr 20;12(4):370. doi: 10.3390/insects12040370 (PMC8074895; doi:10.3390/insects12040370)
Supplement: Supplementary file 1 [file insects-12-00370-s001.zip › insects-1129052-Supplementary.pdf]

Table S1 Sequences and relevant information used for phylogenetic analysis of the trehalase genes

Genes name	GenBank No.	Species	Phylogenetic group
AaTre2	XP_021705796.1	*Aedes aegypti*	Diptera
AgTre2	XP_320471.4	*Anopheles gambiae*	Diptera
AglTre1	AFJ00065.1	*Aphis glycines*	Hemiptera
AmTre1	XP_393963.3	*Apis mellifera*	Hymenoptera
AmTre2	NP_001106141.1	*Apis mellifera*	Hymenoptera
DmTre2	ABH06695.1	*Drosophila melanogaste*	Diptera
BmTre1	BAA13042.1	*Bombyx mori*	Lepidoptera
BmTre2	BAE45249.1	*Bombyx mori*	Lepidoptera
LmTre1	ACP28173.1	*Locusta migratoria manilensis*	Orthoptera
NlTre1	ACN85420.1	*Nilaparvata lugens*	Hemiptera
NlTre2	ACV20872.1	*Nilaparvata lugens*	Hemiptera
OmfTre1	ABO20846.1	*Omphisa fuscidentalis*	Lepidoptera
OmfTre2	ABO20845.1	*Omphisa fuscidentalis*	Lepidoptera
SeTre1	ABY86218.1	*Spodoptera exigua*	Lepidoptera
SeTre2	ABU95354.1	*Spodoptera exigua*	Lepidoptera
SfTre1	AQS60672.1	*Sogatella furcifera*	Homoptera
SfTre2	AFO54713.1	*Sogatella furcifera*	Homoptera
TcTre1	XP_973919.1	*Tribolium castaneum*	Coleoptera
TcTre2	XP_972610.2	*Tribolium castaneum*	Coleoptera
HaTre1	AJK29979.1	*Helicoverpa armigera*	Lepidoptera
HaTre2	AJK29980.1	*Helicoverpa armigera*	Lepidoptera
HvTre1	AZM68711.1	*Heortia vitessoides*	Lepidoptera
HvTre2	AYO46921.1	*Heortia vitessoides*	Lepidoptera
SlTre1	ADA63846.1	*Spodoptera litura*	Lepidoptera
SlTre2	ADA63845.1	*Spodoptera litura*	Lepidoptera
CmTre1	ALF03966.1	*Cnaphalocrocis medinalis*	Lepidoptera
CmTre2	ANC68249.1	*Cnaphalocrocis medinalis*	Lepidoptera
GpTre1	MN915101	*Glyphodes pyloalis*	Lepidoptera
GpTre2	MN915102	*Glyphodes pyloalis*	Lepidoptera
OfTre1	ANY30160.1	*Ostrinia furnacalis*	Lepidoptera
OfTre2	ANY30159.1	*Ostrinia furnacalis*	Lepidoptera

Table S2 Primers used to synthesize dsRNA

Primer Names	Sequence(5'-3')
GpTre1-1-Olig-1	GATCACTAATACGACTCACTATAGGGGCAAGGACTTGAACGGTATTT
GpTre1-1-Olig-2	AA ATACCGTTCAAGTCCTTGCCCCTATAGTGAGTCGTATTAGTGATC
GpTre1-1-Olig-3	AAGCAAGGACTTGAACGGTATCCCTATAGTGAGTCGTATTAGTGATC
GpTre1-1-Olig-4	GATCACTAATACGACTCACTATAGGG ATACCGTTCAAGTCCTTGCTT
GpTre1-2-Olig-1	GATCACTAATACGACTCACTATAGGGGCTCGTGTCTTGGAATATTTT
GpTre1-2-Olig-2	AAAATATTCCAAGACACGAGCCCCTATAGTGAGTCGTATTAGTGATC
GpTre1-2-Olig-3	AAGCTCGTGTCTTGGAATATTCCCTATAGTGAGTCGTATTAGTGATC
GpTre1-2-Olig-4	GATCACTAATACGACTCACTATAGGGAATATTCCAAGACACGAGCTT
GpTre2-1-Olig-1	GATCACTAATACGACTCACTATAGGGGCAGCATGTCAAGGAGAATTT
GpTre2-1-Olig-2	AAATTCTCCTTGACATGCTGCCCCTATAGTGAGTCGTATTAGTGATC
GpTre2-1-Olig-3	AAGCAGCATGTCAAGGAGAATCCCTATAGTGAGTCGTATTAGTGATC
GpTre2-1-Olig-4	GATCACTAATACGACTCACTATAGGGATTCTCCTTGACATGCTGCTT
GpTre2-2-Olig-1	GATCACTAATACGACTCACTATAGGGGCGTCTGGTTAGACTTCAATT
GpTre2-2-Olig-2	AATTGAAGTCTAACCAGACGCCCCTATAGTGAGTCGTATTAGTGATC
GpTre2-2-Olig-3	AAGCGTCTGGTTAGACTTCAACCCTATAGTGAGTCGTATTAGTGATC
GpTre2-2-Olig-4	GATCACTAATACGACTCACTATAGGGTTGAAGTCTAACCAGACGCTT

Table S3 Primers used in RT-qPCR

Gene Names	Forward Primer (5'-3')	Reverse Primer (5'-3')
GpTre1	GAGCCTCGGAAAACATCGCA	CAAGACACGAGCGGAATGCT
GpTre2	ACGGAAACACGATTTCGGCA	CCATCACGACGAGTAACGCC
GpCHSA	TACGCTTTCCACATCACCGC	ACGGGCCTTCTCTTCCTTGT
GpCHSB	ACTTGGCTTTGGGCAGCTTT	GGTCCCTCGTCAACGCATTT
GpCDA1	TTCAAGCCATTCGCTGTCCC	CCAGGAAGCCATCTTGGCAG
GpCDA5	GTGCTTCCCTCCTAACACGC	CCCTTTCGATGGCAGGGTTC
GpCDA2	TTGGTGTGCGTGCTCCTTAC	TATGGGCGTTACCGTTGCAC
GpCHT-h	GCGACCCTTACAGAGGCAAC	TTTTCGCTTCACCGCATCGT
GpCHT3a	TTCAACGACTACAGCCCCGA	GAGAAGTAGCCGTTCAGGCG
GpCHT3b	TCGGGGAGAAGGTCGAGAGA	ATTCCAGGGCCAGTCGCAT
GpUAP	ACAACGTCGGTTTACCCTCC	CAGTTGGAGCCATTGTGTGC
GpPAGM	ATCAAGCAGGTGAACGCAGA	CGTGGTGACGATACCGAACT
GpCDA4	GCCCTATACCAACAACGCCC	AGAAAGTCCCGCGTATGGGA
GpCHT7	GGAGGAGTCTGTGGTGGGAA	ACGCTACTGAGGCCCAATCT
GpRpl32	CGATCACCTTCCGCTTCT	TGCTACCCAATGGCTTCC

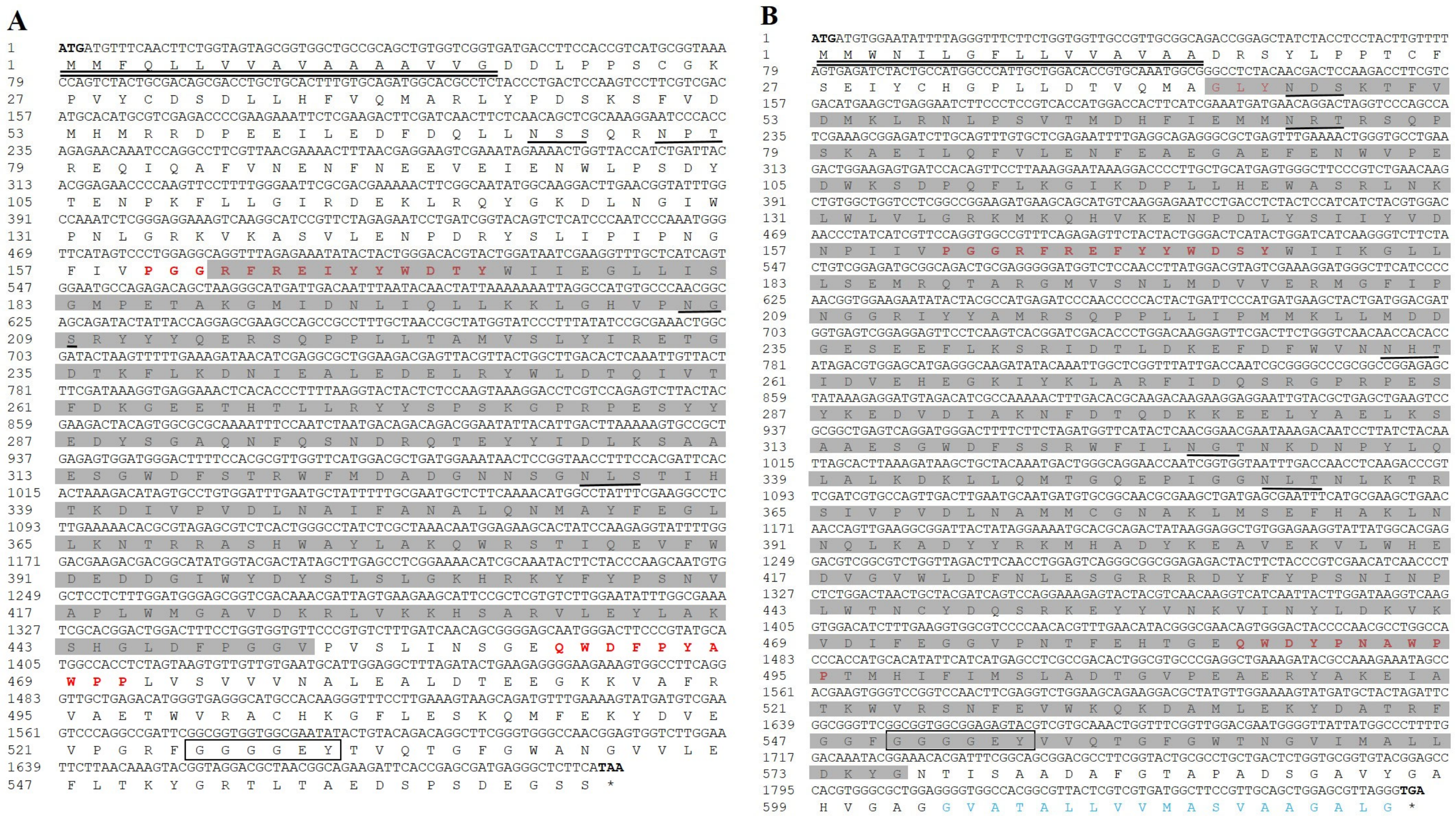

Figure S1. Characteristic analysis of GpTre1 and GpTre2 sequence. (A) and (B) are GpTre1 and GpTre2 nucleotide and deduced amino acid sequences, respectively.

Underlines are potential N-glycosylation sites predicted by PROSCAN. The gray background is the amino acid sequence of the putative catalytic domain. The red letters are signature sequences that might be involved in catalytic function. Double-underlines are signal peptide. The boxes are highly conserved glycine-rich regions. The blue letters are the putative transmembrane region predicted by TMHMM Server v. 2.0.
